# Essay on the Pathology of the Blood

**Published:** 1844-01

**Authors:** 


ART. VII.

*Essai D'Hématologie pathologique.* Par G. ANDRAL, Professeur de Pathologie, &c. à la Faculté de Médecine, &c.—*Paris,* 1843. 8vo, pp. 186.

*Essay on the Pathology of the Blood.* By G. ANDRAL, M.D. &c.—*Paris,* 1843.

IN 1840 and 1841, M. Andral read before the Académie des Sciences two memoirs, containing the results of researches on the pathology of the blood, in which he had been assisted by M. Gavarret. These memoirs were subsequently published in the 'Annales de Chimie et de Physique,' tome lxxv, and in tome v, 3e serie, of the same work. The latter memoir was confined to the blood of certain domestic animals in health and disease, the former to the blood of man in the like conditions. The present essay professes to give merely a further development of the facts and ideas contained in these memoirs, without laying claim to be considered a complete work on the pathology of the blood. The new facts are comparatively few. The new essay, and the former memoir on the pathology of human blood, are neither of them distinguished so much by discovery or real novelty of conception, as by the number and apparent precision of the experimental details on some of the most important points on the pathology of the blood; and by the decided manner in which the views are expressed, which the author has conceived to be deducible from his researches. The facts speak for themselves, and will always pass at their own unquestionable value. The conclusions, or we should rather say the theories, also speak for themselves; but many of them will certainly not pass current for any length of time; and indeed some of them cannot be admitted at all. They bear the stamp of M. Andral's customary zeal and quick conception; but they betray, in some respects, a defective acquaintance with facts already ascertained, and an indisposition to view the facts in every possible light, before their meaning is determined.

As our object in the present article is to give the result of the author's researches on the state of the blood in different diseases, we must pass over the introductory part of the essay with very brief notice. It is justly observed that we cannot form a correct notion of the healthy state of the blood in man by what we learn of that fluid in the lower animals; and this truth is enforced by the interesting and well-known fact, that what in man would be considered a morbid state of the blood, in certain animals belongs to the physiological or healthy state. Thus the buffy coat is not a sign of disease in the horse. It is proved to be of quite as much importance that the varieties which may exist in the proportion of the different physical elements of the blood to one another in different persons enjoying good health, should be carefully ascertained; for there is a considerable range for each element between the maximum and minimum in which it may be present in the state of health. M. Andral's researches on the blood have been hitherto confined to the proportion in which the globules, fibrin, solids of the serum, and water, may exist in health and disease. The following is his estimate of that proportion in the former state: the fibrin he considers as existing in the healthy blood in the mean proportion of 3 parts in 1000. Without any disturbance of the physiological condition, it may be found either so low as 2·5 or 2 per 1000, or as high as 3·5, or even 4; but the two extremes now mentioned are said to be rarely found in health, and should be considered as exceptions dependent on idiosyncrasy. The mean proportion of the globules is fixed at 127 parts in 1000; the extremes in the physiological condition of the system being 140 and 110—the former proportion verging towards the condition of the blood characteristic of plethora. The solids of the serum are estimated at 80 parts in the 1000, and the water of the blood at 790. These mean proportions M. Andral states to be the same as those previously adopted by Lecanu and Dumas, on whose authority it would appear that he has chiefly relied for the proportion of the several elements in question in the normal condition of the economy.

As the importance to be attached to the results of researches, in order to determine whether any, and what, changes occur in the elements of the blood in disease, depends very much on a previous determination of the condition of those elements which is compatible with the state of health, we cannot help regretting that M. Andral does not furnish us in this work with such details, resting on his own authority. If deviations from a healthy condition of the system, even of no very remarkable intensity, be capable of altering the constitution of the blood, the necessity of having ascertained, with scrupulous accuracy, the precise condition of those individuals from whom the healthy standard of the blood professes to be determined, is obvious to all, and we are far from being assured that this amount of accuracy has been attained by those on whose authority so much reliance is placed. The pertinence of this remark will appear sufficiently obvious, when it is considered that chemists fully as well entitled to judge of the medical questions involved in an inquiry of this sort, differ very materially from Lecanu and Dumas in their estimate of the proportion in which the several substances which have been mentioned exists in the blood. Thus, Denis conceives that the mean proportion of globules is 152 per 1000; Letellier that the fibrin ranges between 1·6 and 4·3; Lassaigne fixes it at 1·2; Fourcroy nearly coincides with Letellier, stating its range to be between 1·5 and 4·3. In regard to the serum, a discordance quite as considerable exists; for while Andral, and the authorities in which he confides, maintains that the serum holds 80 parts of solid matter in solution, of which 8 are saline matters and the rest albumen, Berzelius has found the albumen to amount to 80, and the saline and extractive matters to 14; Marcet the albumen to 86, and the other matters to 13. Andral does indeed allow, that in respect to the albumen and other matters of the serum, there are certain varieties in the proportion in which they may occur still compatible with health, but that there is a particular deficiency of albumen which he has never witnessed except in disease, of which more hereafter.

We cannot place any great confidence in the microscopical researches of M. Andral, on the structure and peculiar appearances of the blood-corpuscle; since it is evident to us that he is not sufficiently qualified for the inquiry, either by skill in the use of the instrument, and in the interpretation of the appearances presented by it, or by knowledge of what has been done by other microscopists. Thus we find him maintaining the extraordinary position, that the well-known granular or mulberry appearance is produced in the blood-corpuscle by the adhesion to its surface of the molecules suspended in the fluid ; disregarding the fact that the diameter of the granular or mulberry corpuscles is smaller than that of the normal blood-discs. And we afterwards encounter the completely untenable doctrine, that the fibrin of buffy blood exists in the state of colourless corpuscles, the gradual coalescence of which gives origin to the fibrous network which it is seen to present. It seems very certain that he has committed a serious and unaccountable mistake in having failed to recognize the fibrin as, whatever other form it may assume, existing in the liquor sanguinis in a state of solution, just as albumen does; from which it differs in its property of spontaneously coagulating when in a state of rest.

Plethora. The author commences the pathological portion of his work with an account of the blood of plethora. The common impression is, that in a plethoric state of the system the blood is both richer in its organic elements, and more abundant, than in health. Andral observes, that the latter assertion does not admit of being demonstrated, and he undertakes to prove that the former is true only in a sense restricted to one of the organic elements. We doubt if he is justified in confining the peculiarity of the blood in plethora to a mere alteration of proportion in its elements; for while it is true that we have no means of determining the precise amount of blood contained in an individual at a particular time, which is the objection started by Andral to the assertion that the quantity of the blood is increased, we must, notwithstanding, maintain that there may be sufficient grounds for believing that there is an excess of blood in the vessels, though the exact particulars of that excess cannot be ascertained. A preternaturally turgid state of the vascular tissues, unusual fulness and force of the pulse, feelings of over-distension, constriction, or oppression in the head or chest, and the immediate relief afforded by spontaneous or artificial loss of blood, do appear to warrant the general belief that the quantity of the blood has become, under such circumstances, excessive; and it is scarcely fair to found a denial solely on the ground that the amount of that excess cannot be stated in ounces and drachms. The following is M. Andral's account of the constitution of the blood, to which he would refer all the ascertainable phenomena of plethora:

> "Analysis has shown me, in the first place, that it is not true that in plethora, the blood contains notably more fibrin than in any other circumstances; I have found in fact 2·7 of fibrin as the mean proportion of this principle in thirty-one bloodlettings practised on individuals in whom plethora was very marked. Some of them did not yet present notable symptoms; the bloodlettings in them were simply precautions; in the others vertigo, tinnitus, palpitations of the heart, extreme difficulty of breathing, almost apoplectic injection of the conjunctivæ, and of the face, &c., were present. Yet, in these individuals, the fibrin had not attained its physiological mean." (p. 43.)

On this last circumstance, however, he does not insist, but contents himself with affirming that an increase of fibrin is not characteristic of plethora, and that the proportion of it does not, in the majority of cases, attain the highest amount of what has been fixed as compatible with perfect health. In like manner the organic elements of the serum have been found by him unchanged in their proportion to any remarkable degree.

"The globules then remain, and it is actually the great elevation of their standard which establishes in the blood the character of plethora; on the thirty-one bloodlettings of which I have spoken, I found the mean of the globules to be 141; the minimum 131; the maximum 154. The blood then of plethora differs from ordinary blood by the much smaller quantity of water which it contains." (p. 44.)

This condition of the blood he conceives to be the true and only source of certain phenomena hitherto believed to be dependent upon causes of another kind.

"The individuals whose blood contains a superabundance of globules are subject to certain special symptoms, of which probably no very satisfactory explanation has hitherto been given. Thus, the vertigo, dimness of vision, *tinnitus aurium*, heat of the head, which they experience, have been accounted for by congestions of blood towards the brain; but these congestions have never been, in such circumstances, anatomically established, and the passage of blood having an excess of globules through the vessels of the brain appears to me of itself, a circumstance sufficient to account for the symptoms; but, singularly enough, should it happen, on the contrary, that the globules in too small a number traverse these vessels, analogous effects still occur, so that a quantity of globules, either too considerable or too small, disorders in the same manner certain cerebral functions." (pp. 46-7.)

It must be allowed that M. Andral is very easily satisfied. On what ground is it that he considers the mere passage of a superabundance of globules through the brain as a satisfactory explanation of the phenomena referred to? He does not, and we suspect cannot, adduce a single reason for this belief. We acknowledge that general congestion of the vessels of the brain has not been established by anatomical researches as an actual occurrence, but it does not therefore follow that we are obliged to recognize an excess of globules in the blood as the only alternative cause of the phenomena, as Andral seems to suppose. He overlooks altogether the influence of *pressure* on the functions of the brain, an agency which we think must be admitted to be a far more intelligible source of the disturbances in question. A tight neckcloth, or an inverted posture, will cause both the phenomena mentioned, and an increase of pressure on the brain, but we see no reason to presume that either will produce an increased transmission of the blood globules through that organ. That such an increase of pressure does occur in the case of persons presenting the phenomena referred to, is highly probable from the frequency of the complication of apoplexy with diseases of the heart, in which we know there exists no excess of globules, and from the large quantity of blood not unfrequently witnessed after death from apoplexy in plethoric persons in the integuments of the head, and in the face, indicative of what we must take the liberty of still terming, in the want of a better expression, determination of blood to the head.

Then, as to the production of symptoms of the same sort by an opposite condition of the blood, its deficiency of globules, there are not less valid reasons for conceiving rather that a diminution of pressure on the brain is an adequate cause of such symptoms, than that they depend on poverty of the blood in globules. Witness the effects of a low position of the head in removing the tinnitus, vertigo, and indistinct or troubled vision, which indicate the approach of syncope. That this is the more probable explanation of the origin of similar symptoms in chronic cases, in which the blood is actually deficient in globules, appears from the circumstance that a posture by which increase of pressure is ensured, operates in the same way on them as on those of syncope. An instructive example of this is afforded by the case related by Dr. Abercrombie of a gentleman who had become anemic from extensive depletion and abstinence, and in consequence was affected with deafness, and in whom the interesting circumstance was ascertained that when he stooped so low as to produce flushing of the face, his hearing became distinct. What arguments or facts can be adduced to render it probable that the condition of the blood in respect to the quantity of the globules affords an explanation preferable to those we have now mentioned, we know not; and in the utter absence of any, we leave the reader to form his own estimate of the naked theory of M. Andral. We do not wish to deny, however, that the blood of plethoric persons is usually characterized by an excess in the proportion of red corpuscles; and the facts stated by M. Andral, in reference to the diminution in the amount of these, always produced by loss of blood, harmonize well with the well-known beneficial effect of bloodletting in this state of the system.

Anemia. In anemia, which is next treated of, an opposite condition of the blood exists—it is defective in globules. This state of the blood may originate under various circumstances. Privations, protracted diseases of various kinds, loss of blood, and the constitutional affection which issues in chlorosis, are all liable to induce a greater proportional diminution of the globules than of the other elements of the blood; and sometimes a remarkable diminution of the former without any appreciable decrease of the latter. All this has been long known; but we are indebted to M. Andral for some interesting and precise details illustrative of the facts. He divides anemia into the incipient and confirmed. In the incipient he found the blood in sixteen cases to possess the average amount of globules of 109 parts in the 1000; and in the confirmed he found the average of twenty-four cases to be 65 in the 1000. These were all cases of what he terms spontaneous anemia, and in this form of the disorder, the lowest amount of the globules that he has yet noticed was 28 in the 1000. He conceives it to be of importance to distinguish between the several kinds of anemia, in determining the composition of the blood. Thus, in the spontaneous anemia, whether slight or extreme, he found the globules alone diminished; the fibrin and the solids of the serum maintained their normal standard, the sixteen cases of slight anemia having presented, as a mean of fibrin, 3·0; and the twenty-four cases of confirmed anemia a mean of 3·3. To this head of spontaneous anemia pertains chlorosis, the disorder which affords the most familiar example of the anemic state, when by this term is meant a deficiency of the globules to so great an amount as to reduce the blood to a pale colour. Another kind of anemia is that which succeeds loss of blood, and it may consist of two conditions of that fluid, either of a simple diminution of the globules, which is the first effect of every hemorrhage, or of a diminution also of the fibrin and albumen of the serum. In illustration of the latter, the author mentions an instance of a female who had experienced very abundant hemorrhages from the uterus, and whose blood contained only 21 in the thousand of globules, 1·8 of fibrin, and

61 of solid matters of the serum, the proportion of water having amounted to 915. Anemia may also be the result of certain appreciable modifications of the organism which exerts an influence on the blood. In that case the composition of the blood is the same as in spontaneous anemia, the globules only are diminished. Examples of it are furnished by the blood of pregnant females, in whom the blood loses some of its globules without parting with any of its fibrin. The average of the globules in pregnancy was found to be the same as of incipient spontaneous anemia, 109. Though females are much the most liable to anemia, for reasons with which we are quite unacquainted, it is yet well known that anemia in any of its forms may occur in males also, the spontaneous occurring with all the symptoms which characterize chlorosis in the female, and with an alteration of the blood of exactly the same sort, a decrease in the globules alone. In the cachectic state which follows the prolonged influence of lead, M. Andral has found the same condition of the blood as in the spontaneous anemia, the fibrin and solids of the serum remaining in their normal proportions.

Examined by the microscope, it has appeared to the author, in two cases of chlorosis, that the globules had become much smaller than ordinary, and, at the same time, had no longer their accustomed figure; they were apparently broken, and diffused in the form of fragments over the field of the microscope. This aspect of the globules ceases as the chlorosis becomes cured, and the author has had occasion to notice the normal characters of the globules thus restored, two months after they had exhibited the alterations in question, the patient having passed from a chlorotic into a state of actual plethora within that period.

It has been long known that the blood, in chlorosis, is liable to present a buffy coat, the reason of which is thus stated by M. Andral:

"It is because the blood of the chlorotic has poisoned all its fibrin, and has lost part of its globules; and consequently there is actually in the blood, as in that of inflammations, or as normally in the blood of certain animals, an excess of fibrin in relation to the globules; now, whenever this excess occurs, whether it be relative or absolute, when at the same time the coagulation of the fibrin is not correspondingly rapid, we perceive this substance accumulate at the surface of the clot, and the buffed appearance to be produced. Hence the reason why the blood of anemic persons may be buffed, and that of the plethoric is not; hence, also, the reason why in the former the crassamentum is more firm and dense than in the latter." (p. 35.)

In reference to the functional disorders which accompany anemia, he states that they correspond in intensity to the degree of diminution in the number of the globules; and he labours especially to prove this, in regard to the murmur which is heard in the heart and arteries. The following are the deductions which he draws from his observations on this subject;

"1. When the globules are so few as to be below 80, the bellows-murmur exists in the arteries as a constant occurrence. I have not found a single exception to this law.

"2. When the globules are above 80, the murmur may still occur, but it is not constant; we continue to hear it pretty often when the globules range between 80 and 100; it happens still, but much less frequently, when the globules exceed 100; and we never observe it, allied at least to an alteration of the blood, when the amount of the globules is elevated above its physiological mean.

"Whatever, in other respects, may be the nature of the disease in which the diminution of the globules exists, the bellows-murmur in the arteries does not the less occur. I have found it in cases of the most different characters, putrid and eruptive fevers, pneumonias, articular rheumatisms, and in a great many chronic maladies. But, in all these cases, it happened only in connexion with the state of the globules indicated above." (pp. 57-9.)

He adds, that the murmur occurs pretty often in pregnant females, and in connexion with the diminution of the globules frequently found in them; and further, that the intensity of the murmur is generally proportional to the degree of decrease in the globules. Thus, in 22 cases of chlorosis, he found the murmur intermittent 8 times, the amount of globules ranging between 117 and 77; and the murmur continued 14 times, the amount of globules ranging between 113 and 28.

Beyond the statement of these particulars M. Andral does not go; and by thus stopping short, we conceive that he leaves the discussion of the source of the chlorotic murmur in an unsatisfactory state. The hypothesis, that the condition of the blood is the only one on which the murmur depends (and this seems plainly the inference to which the writer points,) appears irreconcileable with some of his own statements. The whole scope of the observations in respect to the murmur of the arteries bears simply upon the general fact, that whenever the murmur existed the globules were found diminished; but in order to have established the dependence of the murmur on that diminution he ought to have proved the converse also, that whenever the globules were in like manner diminished, the murmur existed: this, however, is not attempted, excepting to the extent contained in the first of the two rules quoted above. It may also be objected, that, in cases of confirmed chlorosis, there are other alterations in the blood, besides diminution of the globules. This appears from the researches of M. L'Heritier,* who affirms, upon the basis of a series of experiments that seem to have been much more extensive (on this point at least) than those of M. Andral, that *all* the solids of the blood are diminished in such cases.

What we consider of especial consequence in the observations of M. Andral, in reference to the relation between the arterial murmur in chlorosis and the amount of the globules, is, that if the existence of the murmur, and more particularly of its intensity, is adopted as an indication of the degree of the diminution of the globules, and consequently of the state of advancement which the disease has reached, it will be apt to mislead; for though tenuity of the blood is granted on all hands as affording a facility for the generation of the murmur, it is very certain that other causes powerfully concur in its production; and according to their accidental and even temporary intensity the murmur may vary in different cases, irrespective of the amount of change in the elements of the blood. We refer the reader to Dr. Hope's chapters on "Inorganic Murmurs," for most conclusive arguments in favour of the doctrine, that the rate at which the blood moves in the arteries has very much to do with the production and degree of the anemic or chlorotic murmur.

In concluding this chapter on the diminution of the globules, M. Andral takes occasion to notice that he has not found any decrease of temperature attendant on the deficiency of globules. Even when the

* Traité de Chimie Pathologique, pp. 249-50.

globules were as low as 21, he found the temperature in the axilla vary from 37° to 38° (cent.); and when febrile action became established in such cases the temperature rose as usual.

FEVERS. The next article, on the blood in fevers (pyrexiæ,) we consider the most valuable—in so far as the author's own researches are concerned—in the whole work. It derives its value in an eminent degree from the contrast which its details present to those contained in the article which follows, on the blood of inflammations. We extract the first two paragraphs entire, containing, as they do, a succinct, luminous, and not less truthful expression of important pathological views of the class of diseases to which they refer.

"The *pyrexiæ* form a grand class of acute diseases which vain attempts have been made to blot out from the tables of nosology, in order to rank them in the order of inflammations. Such an attempt ought not to be encouraged; the pyrexiæ exist as distinct diseases, (*maladies à part,*) the causes which often develop them, the symptoms which characterize them, the special nature of the alterations which they effect on the solids, the epoch of development of these alterations often posterior to that of the febrile action, are so many serious reasons for not confounding them with inflammations ; but analysis of the blood tends besides to establish a difference, the most remarkable between the one and the other class. The results furnished by this analysis have something so marked, that they seem to me to determine in a definite manner the distinction, vainly opposed, of fevers and inflammations ; it is this which I proceed to demonstrate.

"Whilst in inflammations, there are always two constant alterations which advance together, that of a solid and that of the blood, there is nothing of the kind in fevers ; in these diseases indeed, the only phenomenon which is never wanting, is the fever itself; the alterations, various besides, of which the solids are the seat, may be completely absent, neither do the changes of composition which analysis has discovered in the blood, show themselves in every case ; so that in the actual state of our knowledge, the character of fevers remains still a negative one ; that is to say, that, to the best informed, the fever, which accompanies the *pyrexiæ,* does not acknowledge either in the solids or in the blood, any alteration which can account for it. At the same time, in the solids and in the blood, we can more or less frequently discover alterations ; but they are only the effects of a cause more sudden which reigns in the system, effects, nevertheless, which it is important to study well, since in their turn they become themselves the cause of a certain number of symptoms, and because by their seat and nature they serve to class and to denominate the *pyrexiæ.*" (pp. 61-2.)

The general fact of there being a tendency throughout the progress of fevers, to a reduction in the solids of the blood, we need scarcely observe, has been long known in this country. The observations of Dr. Reid Clanny on this subject, imperfect though they be, may be noticed as having pretty generally diffused among us a belief that such is the constitution of the blood in continued fever; but M. Andral's researches have brought to light not a few additional facts of much importance and interest, and have placed the whole subject on a very different footing, in regard to precision and pathological value, from that in which he found it.

It is doubtless well known to our readers that in inflammations the proportion of the fibrin of the blood is increased in relation to the globules. Now in fevers, the very opposite condition is exhibited, to this extent at least, that the fibrin invariably decreases, and even though the globules do so to a considerable amount, at the same time their proportion to the fibrin in the diminished state of both, is always much greater than in health. In the early stage of the fever it sometimes happened that the difference in the relation of the two elements to one another was rather owing to the increase of the globules than to the degree of the diminution which the fibrin had actually undergone. Thus the blood in some cases exhibited an increase of globules to between 130 and 149; but in general, and always in the advanced periods of the fever if uncomplicated, the difference was due to the decrease of the fibrin. This tendency to the decrease of the fibrin in fevers is arrested in the event of intercurrent inflammation, though the ensuing increase of the fibrin, (for it may rise in the course of these intercurrent inflammations,) is never so considerable as when the inflammations occur independently of the fever. And it is most interesting to notice that the occurrence of those specific inflammations which are proper to, and characteristic of the fever, has not the effect of thus augmenting the fibrin. The pustular inflammation in smallpox, the inflammatory efflorescence on the surface in measles and scarlet-fever, are not accompanied by an elevation of the fibrin above the natural standard. In smallpox, as appears from his former memoir, M. Andral found the proportion of fibrin, only once somewhat elevated, but even then the total amount was 4·4, scarcely, if at all, beyond the maximum limit of health; and the common range of it was from 3·5 to 2; yet in one case it had fallen so low as 1·1. Something like the ordinary effect of inflammation was noticed occasionally in smallpox; thus when a first bleeding had discovered a low proportion of fibrin, a second practised while the disease was still advancing, has discovered an increase on the former proportion. In one case, for instance, a first bleeding revealed the proportion of fibrin to be 1·1, a second to be 2, and in another case the increase was from 2·6 to 3·5. In both these instances, the progress of the change was, as in simple inflammations, but the energy of the cause, whatever it may be, which accomplishes in inflammation the increase of the fibrin, seems to have been too feeble in these cases of smallpox to overcome the peculiar influence which has the power in fevers of depressing the proportion of that element of the blood.

Inflammations. In the fourth article the changes which the blood undergoes in inflammations are described. M. Andral's researches on the effect of inflammation on the blood, are interesting chiefly on account of the details which he furnishes of the amount to which the fibrin may increase in different inflammatory affections. It is scarcely necessary to mention that the most characteristic alterations which the constitution of the blood undergoes in inflammation consist of a gradual decrease in the proportion of the globules, and an increase in that of the fibrin. The albumen of the serum too, sometimes, but not always, has been found affected, the tendency of the inflammatory process being to increase the amount of it. It was in acute articular rheumatism, and in acute pneumonia, that the greatest increase of the fibrin was discovered. The mean increase in these two diseases fluctuated between 7 and 8 parts per thousand; the extremes were 4, and 10·5. In acute bronchitis, pleurisy, peritonitis, tonsillitis, erysipelas, and other inflammations, the operation of the general law was abundantly manifest; but in none of these did the increase of the fibrin reach the same amount as in the two diseases we have specified: the common augmentation in the other affections having been 5 and 6 parts only; and the highest amount did not exceed 9·3; an elevation which was noticed only once, and that in a case of acute bronchitis.

The following is a summary of the results obtained in the experiments on the blood of inflammation.

"First, since inflammation of the cellular tissue is regarded as the type of inflammation generally, I shall speak of a case of phlegmon of small extent affecting one of the legs, which terminated in ulcers, and which was accompanied with but a slight febrile movement. In a first bloodletting we found the fibrin 4·7, and in a second 5. In another case of phlegmon, which existed in the mamma, and which terminated by resolution, the blood furnished 4·5 of fibrin during the continuance of the slight inflammation, and 3·7 only, some time after it had terminated.

"Among 84 bloodlettings practised during the course of well-marked pneumonia, there were 7 only in which the fibrin fluctuated between 4 and 5; in the 77 others it surpassed this amount, maintaining itself eleven times between 5 and 6, nineteen times between 6 and 7, fifteen times between 7 and 8, seventeen times between 8 and 9, nine times between 9 and 10, and six times elevating itself to 10, or exceeding a little this last degree.

"In inflammations of the mucous membranes we found the fibrin retaining its normal quantity if these inflammations were slight, of small extent, and without fever; but whenever they had a certain degree of intensity, and when febrile action accompanied them, the fibrin invariably increased.

"Thus I have seen it attain the amount of 6·7 and 9 in cases of bronchitis which had much extent and acuteness.......... In inflammations of the mucous membrane of the digestive passages, the same increase occurred." (pp. 86-7.)

M. Andral states, however, at p. 70, that, though whenever a portion of the digestive mucous membrane, "from the fauces, including the tonsils to the end of the colon, is seized with the inflammatory process sufficiently acute to produce fever," there is a notable increase of the fibrin; yet the elevation amounts only to 5, 6, or 7, and never higher.

Having found in four cases of mercurial stomatitis the occurrence of the same condition of the blood as in the simple, or non-specific inflammations, M. Andral remarks—

"Thus mercurial stomatitis, notwithstanding its specific nature, does not differ from ordinary *phlegmasiæ* in respect to the influence which it exerts on the blood; and yet it is often averred that mercury introduced into the economy has the effect of producing in the blood a state of dissolution, which cannot exist with an augmentation of fibrin. It is possible that this may occur after a prolonged employment of this medicine, but it certainly does not during the earlier periods of its administration. Consequently, when it is given to combat certain acute inflammations—peritonitis, for example—one is not entitled to admit that its antiphlogistic action depends on its creating in the blood a disposition the reverse of that which coincides, in that fluid, with the existence of a state of inflammation." (p. 90.)

As long as acute inflammation continues unabated the elevated proportion of fibrin remains unchanged, notwithstanding repeated bloodlettings and the most restricted diet. And, indeed, so constant and powerful is the agent which augments the fibrin in inflammation, that no circumstances of previous debility, or privation, present the characteristic change. This is remarkably exemplified by the results of certain researches of the author, on the effects of starvation on the blood of dogs. He was surprised to find that after a time the fibrin had actually increased in amount, but, he adds, "I ceased to be surprised, when, on examining the bodies of these animals, I found alterations in their stomach of the most decidedly inflammatory nature; such as lively redness, softening, and numerous ulcerations of the mucous membranes of the viscus." By bloodlettings, practised before they were deprived of their food, he found in three dogs the fibrin to amount to 2·3, 2·2, and 1·6, respectively, being the natural range of the fibrine in those animals. The first dog was starved for twenty-one days, after which he died. When bled on the seventh day, the fibrin was found to have risen to 3·9, and on the fourteenth day to 4·5. The second was allowed some water only, and died in eighteen days; on the seventh day the proportion of fibrin was found to be 2·9; and on the fourteenth 4·0. The third animal lived twenty-six days, having been supplied every morning with a very small ration of soup. The blood, on the seventh and fourteenth days, exhibited no considerable change, the fibrin having increased only to 1·8. But on the twenty-second day, when the insufficient nourishment had an opportunity of affecting the economy for a longer time, it was far otherwise, for then the blood showed an increase of the fibrin to 3·3. In this instance, the amount of disease in the stomach was less considerable than in the others. (pp. 82-3.) These facts, concerning the state of the stomach in animals long deprived of food, accord with what Hunter conceived to be the effect of starvation on that viscus. The increase of the fibrin is confined to acute and subacute inflammations, is considerably less in the latter than in the former, and does not occur at all in chronic inflammations.

The elevation in the quantity of the fibrin appears in the blood from the very commencement of the inflammation. M. Andral has had occasion to bleed on the morning of the day in which inflammation began, before its actual invasion too, and again a few hours after the process had begun; and in the blood of the former evacuation he has found the fibrin in its normal quantity, or that of the latter in excess. "Hitherto," he says, "I have endeavoured in vain to determine whether, before the change which denotes the inflammation had appeared in the solid textures, the blood had not become modified in its composition. I have not been able to establish this; and my analyses have not yet shown me anything else than the simultaneous origin of these two conditions." (p. 98.) He conceives, however, that the question is not yet decided; and hints that the very dissimilar circumstances in which inflammations commence, leave room to doubt whether the starting-point of the inflammation is the same in every instance. What occurs in the case of a burn clearly proves that it is consequently to an alteration of the solids that the blood, in that instance at least, becomes modified in its composition; and, he adds, that, inductively considered, we should suppose it to be the same with other inflammations,—a conclusion which the analyses hitherto made of the blood tend to confirm. The connexion which the inflammatory fever has with the increase of the fibrin appears to him also a matter for further inquiry;—he seems in the text to be struck with the coincidence between the increase and diminution of the former with those of the latter, and yet, in a note acknowledges the impossibility of explaining thus the phenomena of fevers in general. The coincidence is, he might have added, deprived of much, if not of all, of its apparent significancy, by the fact that the conditions of the fibrin and of the fever are intimately related to the extent and intensity of the local inflammation. On this point, however, it must be granted that sometimes the phenomena of the fever are actually present before the local symptoms of the disease have occurred; and are probably therefore occasionally due to the general operation of the cause which, in its ulterior effects, superinduces the local affection. It would be interesting to learn whether in such cases, and before the occurrence of the local disease, the fibrin is actually increased: we know that a buffy coat on the blood is not at that period a characteristic or usual appearance. Again, every physician has met with cases in which the fever has continued, although the blood has ceased to exhibit the buffy coat, and that too in acute inflammation; and M. Andral, at p. 94, records a case of peritonitis which had passed from the acute into a somewhat chronic state, without, however, the fever having ceased for an instant; and yet the fibrin had fallen to 3·5.

In reference to the buffy coat, M. Andral recognizes three conditions which may favour its occurrence;—a great decrease of the globules while the fibrin remains undiminished; an actual increase in the quantity of the fibrin; and a slower coagulation of the blood. The first is the cause to which he assigns the occasional occurrence of the buffed appearance in the blood of chlorosis, in which the globules, in consequence of their diminished number, have a facility for subsiding, and thereby leave the fibrin free to rise to the surface of the crassamentum. The last two he conceives to be the causes of the buffy coat of inflammatory blood. He has completely overlooked another and, perhaps, a more important reason of the buffy coat of inflammatory blood,—the unusual tendency of the globules and fibrin to separate. This is a fact so well ascertained that the omission of all notice of it is not a little singular. More than twenty years ago Dr. John Davy directed attention to the fact, that in inflammatory blood the separation of the fibrin from the red globules may be noticed to occur within the time usually necessary for the coagulation of healthy blood; that, in fact, it may be witnessed in the form of a supernatant liquid stratum within one or two minutes after the blood has been drawn. Schroeder van der Kolk, too, has conclusively demonstrated the same fact; and besides drew, long ago, attention to a remarkable proof of this tendency of the globules and fibrin to separate early, which is exhibited when the blood is spread in a thin layer either on the lancet or any other flat surface. The appearance which is then presented is quite characteristic of inflammatory blood, and proves a disposition in the globules and fibrine to separate altogether, irrespectively of the influence of the gravitation of the globules. The thin layer in question speedily presents a mottled or areolar aspect, due to the red globules being grouped in particles and lines, having the transparent liquid fibrin interposed. Microscopic examination of the habitude of the red globules of inflammatory blood, immediately after it is drawn from a vein, explains the manner in which this appearance is effected. We must refer to Mr. Wharton Jones's Report in our Number for October, 1842, for details connected with this and some other interesting particulars concerning the blood of inflammation, and at present content ourselves with stating, that the globules appear in such blood to have an increased attraction for one another, and that they accumulate almost immediately, on coming into a state of comparative rest, into nummular piles or strings,—an arrangement which of course amounts to a cessation of their state of intermixture with the *liquor sanguinis.* We are aware that it has been supposed that the mottled aspect of the blood in question is owing rather to a repulsion between the globules and the fibrin, but of this there is no proof; while of the increased attraction of the globules for one another there is ocular demonstration.

Pregnancy. In connexion with the account of his researches on the blood of inflammation, M. Andral notices the influence of utero-gestation in elevating the proportion of the fibrin. Having analysed, with M. Gavarret, the blood of thirty-four pregnant females, he has found that from the first to the end of the sixth month the quantity of the fibrin is always below the physiological mean; the mean proportion of the fibrin during these six months having been 2·5; its minimum, 1·9; its maximum, 2·9. But, during the last three months of pregnancy, the mean of the fibrin was found above the physiological standard; it approached 4, and presented a maximum of 4·8. In the last month of pregnancy the increase of the fibrin appeared to have attained its greatest amount, the mean having been 4·3. To this increase of the fibrine, along with the diminution of the globules, he ascribes the occasional occurrence of the buffy coat on the blood of pregnant females. The diminution of the globules is so general, that, among the thirty-four females, he found these bodies to range between 120 and 95 in twenty-six cases; and between 125 and 120 in six others.

"The blood then manifests a remarkable tendency to assume the character of the blood of inflammations, and without doubt we have to reflect on the relation which may exist between the kind of modification which the blood then undergoes, and the development of those special accidents—generally of an inflammatory appearance—which affect so often females recently delivered. Ought we to regard the slight excess of fibrin which, in them, exists in the blood, as a predisposing cause of these accidents?" (p. 104.)

This question, in connexion with the general one of the changes which the blood undergoes previously to the actual occurrence of inflammation, he will soon endeavour to determine.

Action of pus on the blood. Few of the author's observations on pus are worthy of being specially noticed. The following are, however, of some interest:

"I have ascertained, by experiment, that on mingling pus directly with blood, the latter undergoes no appreciable alteration either in its aspect to the naked eye, or under the microscope, or in its quantity of fibrin, if the pus be still fresh. But it is otherwise if the pus mixed with the blood have existed a long time out of the body, and have already undergone a certain degree of decomposition." (p. 117.) [He mingled the latter sort of pus with the blood of a case of acute articular rheumatism, the pus being in the proportion of one tenth of the mixture.] "An hour after the mixture had been made I found the clot well formed, but without the buffy coat, and surrounded with a transparent serum. The pus, putrefied as it was, appeared then to have no particular action on the blood, if it be not that it opposed the formation of the buffy coat; it had not altered its consistence. But at the end of twenty-four hours matters were much changed; the vessel was then filled with only a liquid matter, red, and similar to that which is obtained by a mixture of blood and ammonia. . . . . . . Examined by the microscope, the liquid mass presented no longer a trace of blood-globules, but a great many globules of pus were found; so that the putrefaction had not destroyed these." (p. 119.)

"It results obviously, from these experiments, that the influence which the pus exercises on the blood is far from being the same, according as the pus is fresh, or has existed so long out of the living body as to have become putrid. Fresh pus has no appreciable action on the blood; putrid pus acts on it in the same way as ammonia does; it destroys at once the globules and the fibrin. But, what is remarkable, this destruction does not happen immediately; and it is necessary that some hours should elapse of contact between them before the blood begins to present traces of alteration." (p. 120.)

Hence the author concludes, that when pus circulates in the blood the effects on this liquid will depend on the qualities of the pus; and that it is not, in the event of pus altering the blood, its globules but the ammoniacal product which is formed at the expense of the pus itself, which acts injuriously on the blood. He also concludes that it is this ammoniacal product which is transferred on the scalpel from putrefying bodies, and causes those changes which are noticed in the blood, and those symptoms which follow, when punctures have been received with the poisoned instrument; symptoms of the same sort as those which are produced by admission of pus, in certain states, into the circulation. In short, it is to this product that he ascribes the typhoid symptoms which supervene under these circumstances; and the absence of the usual effect of inflammation, when this happens to be present at the same time, or of the fibrin, is ascribed to the operation of the same agent.

HEMORRHAGE. The fifth article, on the state of the blood in hemorrhages, is a long one, but, as much of it is occupied by references to authors, and reasonings in favour of a certain view which the author entertains of the nature of the blood in this morbid condition, what we have to notice, as comparatively new and interesting on this head, admits of being very briefly stated.

"The diminution of the fibrin in relation to the globules is the grand condition of the blood which favours the production of hemorrhages; the relation of these two facts is so constant, that it appears to me impossible not to regard the one as the cause of the other. And that it need not be maintained that it is the hemorrhage which produces in the blood the diminution of the fibrin, it suffices to observe, that it is necessary that the loss of blood should be very abundant in order to produce this effect, and that I have seen the fibrin diminished in these cases, when the quantity of blood lost could certainly not account for it. I add, as an argument which seems to me unanswerable, that if it were the hemorrhage which produced a change in the composition of the blood, we should certainly find the globules diminished in a proportion yet greater than the fibrin; but this not only does not occur, but the globules are the most commonly then in excess compared to the fibrin." (p. 127.)

He mentions two states, of a different kind, in which the law still holds good of a diminution of the fibrin in relation to the globules, which may favour the production of hemorrhages. In the one the only change occurs in the globules, which have become elevated above their normal amount, and hence the hemorrhages of plethora. In the other the fibrin alone is essentially altered, and has become less abundant than usual. This is the condition of the blood in scorbutus. He mentions an instance in which he had an opportunity of examining the blood of scorbutus. The trunk, the face, and the limbs were covered with petechiæ, and large ecchymoses; the gums were soft, swelled, and bleeding, and epistaxis occurred from time to time; the pulse was 60, the head was affected with a sense of weight and vertigo. The blood which was drawn contained

119 of globules, 86 in solids of the serum, and only 1·6 in fibrin. It was not in cases of the general hemorrhagic tendency only that he ascertained the diminution of the fibrin, but also in cases of cerebral hemorrhage.

"Whether the blood escape from several solids simultaneously, or from one only, facts lead to the conclusion that in the second case, as well as in the first, there are hemorrhages which may depend on this,—that the blood, deprived of its normal quantity of fibrin, has lost, by that circumstance, a part of its plasticity; and thus we come to recognize, in an alteration of the blood, the *point de départ* of certain morbid phenomena, the cause of which is the most commonly ascribed to a lesion of the solids. The cases in which the fibrin is only diminished in reference to the globules which are in excess, belong, from their symptoms, to the cases of hemorrhage termed active, and the cases in which the quantity of the fibrin is actually diminished belong to those of hemorrhages termed passive. The attentive study of the alterations of the blood comes, then, to afford a new support to this ancient division, which contains theories we could not admit, but which could not be overlooked by the principles of treatment, or by clinical observation." (p. 134.)

In his memoir, in the 'Annales de Chimie,' M. Andral conjectures that in *purpura hemorrhagica* the fibrin will be found on examination to be very scanty, and the close analogy between that disease and the one he describes as *scorbutus*, renders it probable that the blood in both will hereafter be found to be of much the same characters. The hemorrhages in both have been long conceived to depend on a change in the fibrin, and the opinion would claim assent, were it not that certain observations made by most respectable authors, have furnished facts which are utterly irreconcileable with it. Thus Deyeux and Parmentier found in three cases of *scorbutus* the blood about as rich in fibrin, and the clot as consistent, as in health; the late Dr. Parry, of Bath, found the blood in two cases of purpura, to form a firmly contracted coagulum, covered with a buffy coat; and Dr. Babington, too, in a case which presented many of the symptoms of that disease, or of sea scurvy, found the blood of a like firm character, and also surmounted by a buffy coat. Doubtless, M. Andral would endeavour to get rid of all these cases, as he actually does of the first three, by contending that the amount of fibrin was owing to some intercurrent inflammation. But, allowing the fact to be so, it is not the less a serious objection to his doctrine of the dependence of the hemorrhages on a deficiency of the fibrin; for from whatever source the fibrin was derived in these cases, it was present in, at least, its normal proportion; and in the cases of Parry and Babington, probably in excess; yet, notwithstanding, the hemorrhages occurred. The subject therefore manifestly stands in need of additional investigation.

Dropsy. The facts relating to the blood in dropsies, may be divided into those which are negative and those which are positive. M. Andral passes in review the influence of diminution of the fibrin and then of the globules, in producing dropsical effusions, and finds that neither has any direct concern with their manifestation. If a deficiency of the globules were adequate to the production of dropsy, that symptom would doubtless be common in chlorosis, which it certainly is not: and if a decrease of the fibrin were capable of leading to dropsical effusion, we should expect to find this occurrence in scorbutus and purpura and typhoid fever. Yet there is one condition of the blood, he conceives, which we have a right to regard as entailing necessarily the occurrence of dropsy; namely, a diminution of the albumen of the blood. Those who are acquainted with the researches of Drs. Bostock, Christison, and Babington, do not need to be informed that in Bright's disease of the kidney, when the albumen is present in any considerable quantity in the urine, it is proportionally defective in the serum of the blood, and it is in that disease alone, and dependent on the cause in question, that M. Andral has satisfied himself of the intimate connexion between the deficiency of the albumen and the occurrence of dropsy. The occurrence of the dropsy in the acute stage of the disease, when none of the ingredients of the blood are deficient but the albumen, appears to favour this view of the source of the serous infiltrations, in preference to that of Sabatier and Solon, who ascribe it in general terms to the tenuity of the blood considered as a whole,—a condition of it, however, which is confined to the more advanced periods of the disease. Dr. Christison suggests, that in the acute stage it is the deficiency of the albumen which constitutes the condition on which the dropsy depends; while he seems to concur with the authors mentioned in ascribing the infiltrations of the advanced periods to the general deficiency of solid matter in the blood. But if the deficiency of any of the ingredients of the blood is to be regarded as the cause of the dropsy, it must be that of the albumen; for in other respects the blood in this disease of the kidney, in so far as the amount of its natural organic elements is concerned, has precisely the same constitution as the blood of chlorosis, with the single exception that it is also deficient in albumen, which, according to Andral, does not occur in chlorosis. These facts seem to justify the opinion that it is essentially on the deficiency of the albumen that the dropsy depends in Bright's disease. And yet, with all this appearance of proof in favour of the influence of a diminution of the albumen, we should be sorry that further inquiry should be foreclosed by a general acquiescence in this doctrine—and for various reasons. In the first place, because it is well known that in not a few cases dropsy never supervenes, although the amount of albumen in the urine demonstrates that there must be a great deficiency of it in the blood. Secondly, because the deficiency of albumen in the blood is but one of the alterations which this liquid undergoes in disease of the kidney; and, indeed, it is when the albumen is most deficient, i. e. in the acute stage, that other suspicious alterations are the most likely to exist in their greatest degree, namely, the accumulation of those matters, urea and salts, which are not evacuated with the urine in nearly their natural quantity. And, thirdly, because the degree and extent of the dropsy has not been found to have any direct relation to the degree of the coagulability of the urine, that secretion being sometimes found but slightly coagulable when the dropsy is very great, and, as already stated, sometimes absent altogether when the urine abounds in albumen. We do not think that sufficient attention has yet been bestowed on the amount of the salts of the serum, in dropsy from diseased kidney; and this is the more to be regretted, because it is quite a supposable circumstance that their accumulation in the blood may have some influence on the secretion of the various tissues by their stimulant properties. We have sometimes, also, ventured to ask ourselves the question, whether the great proclivity to inflammatory action which obtains in this disease may not be owing in some measure to this state of the blood.

It is not certain that other conditions of the blood, besides those which occur in cases of diseased kidney, may not be sometimes the sources of dropsy; such, more especially, as result from long-continued privation of nutritious aliment. M. Andral conjectures that in such cases also the albumen of the serum becomes ultimately lessened, and that the deficiency constitutes the pathological condition on which the dropsy depends,—an opinion which he thinks is supported by what occurs in sheep which have been long kept in moist localities and insufficiently nourished. Under these circumstances the albumen of the animals' blood was found to have become lessened, at the same time that the animals became hydropic. We have no details, however, from which to judge of the force of this argument, or of the exact nature of the pathological circumstances in which these animals were found.

Organic and nervous diseases. The two short chapters on the blood of organic and nervous diseases, contain little that is worthy of notice. The general tendency of chronic diseases is to lessen the amount of the globules, thereby producing the anemic aspect characteristic of most of those which materially affect the health. The fibrin is always liable to increase on the supervention of inflammatory action, but is in other circumstances not apt to have its ordinary proportions materially disturbed

---

